# A rate-responsive duty-cycling protocol for leadless pacemaker synchronization

**DOI:** 10.1007/s13534-024-00413-z

**Published:** 2024-08-19

**Authors:** Adrian Ryser, Tobias Reichlin, Jürgen Burger, Thomas Niederhauser, Andreas Haeberlin

**Affiliations:** 1 Department of Cardiology, Inselspital, Bern University Hospital, University of Bern, Freiburgstrasse 20, 3010 Bern, BE Switzerland; 2 School for Cellular and Biomedical Sciences, University of Bern, Mittelstrasse 43, 3012 Bern, BE Switzerland; 3 Institute for Human Centered Engineering HuCE, Bern University of Applied Sciences, Quellgasse 21, 2501 Biel, BE Switzerland; 4 ARTORG Center for Biomedical Engineering Research, University of Bern, Murtenstrasse 50, 3008 Bern, BE Switzerland; 5 School of Biomedical and Precision Engineering, University of Bern, Güterstrasse 24/26, 3008 Bern, BE Switzerland

**Keywords:** Implant communication, i2i, Leadless pacemaker, Dual-chamber pacemaker, Communication protocol, Rate-responsive

## Abstract

Dual-chamber leadless pacemakers (LLPMs) consist of two implants, one in the right atrium and one in the right ventricle. Inter-device communication, required for atrioventricular (AV) synchrony, however, reduces the projected longevity of commercial dual-chamber LLPMs by 35–45%. This work analyzes the power-saving potential and the resulting impact on AV-synchrony for a novel LLPM synchronization protocol. Relevant parameters of the proposed window scheduling algorithm were optimized with system-level simulations investigating the resulting trade-off between transceiver current consumption and AV-synchrony. The parameter set included the algorithm’s setpoint for the target number of windows per cardiac cycle and the number of averaging cycles used in the window update calculation. The sensing inputs for the LLPM model were derived from human electrocardiogram recordings in the MIT-BIH Arrhythmia Database. Transceiver current consumption was estimated by combining the simulation results on the required communication resources with electrical measurements of a receiver microchip developed for LLPM synchronization in previous work. The performance ratio given by AV-synchrony divided by current consumption was maximized for a target of one window per cardiac cycle and three averaging cycles. Median transceiver current of both LLPMs combined was 166 nA (interquartile range: 152–183 nA) and median AV-synchrony was 92.5%. This corresponded to median reduction of 18.3% and 3.2% in current consumption and AV-synchrony, respectively, compared to a non-rate-responsive implementation of the same protocol, which prioritized maximum AV-synchrony. In conclusion, adopting a rate-responsive communication protocol may significantly increase device longevity of dual-chamber LLPMs without compromising AV-synchrony, potentially reducing the frequency of device replacements.

## Introduction

Conventional cardiac pacemakers (PMs) have been effectively used in the treatment of bradyarrhythmias for decades. However, conventional PMs suffer from relatively frequent complications affecting around 10% of patients within two months after implantation [1]. Recently, leadless pacemakers (LLPMs) have been introduced to alleviate these complications, which are mostly related to the leads and the pocket implants [1–3]. These highly miniaturized, self-contained devices are directly implanted into the heart [2, 3]. About 80% of transvenous PM patients are treated with a dual-chamber system, which can provide both atrial pacing and atrioventricular (AV) synchronous ventricular stimulation [4]. Recently, the first commercial dual-chamber LLPM has been introduced [5, 6]. This system consists of two separate devices, one in the right atrium and one in the right ventricle, which synchronize pacing activity via wireless communication [5, 6]. Mean AV-synchrony rates exceeding 96% have recently been reported for this dual-chamber LLPM, demonstrating reliable inter-device communication [6]. However, the continuously active transceivers employed for implant communication, estimated to consume $\sim$ 800 nA under default settings, substantially reduce battery lifetime [5, 7]. Specifically, the projected lifetime of the atrial LLPM (ALLPM) is only 5–7 years for dual-chamber operation [7, 8]. This corresponds to a 35–45% longevity reduction compared to single-chamber operation, where no communication is required [7].

**Fig. 1:**
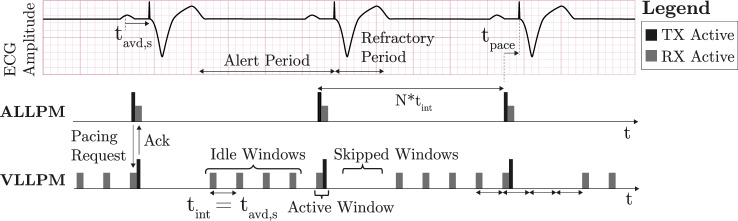
Transceiver activation pattern for LLPM synchronization based on non-rate-responsive synchronous duty-cycling protocol (SDCP) proposed in [9]

Therefore, increasing the longevity of existing dual-chamber LLPMs is highly desirable from a clinical viewpoint. Recent work has proposed a power-optimized communication protocol based on synchronous duty-cycling (SDCP), wherein the transceivers of both implants follow concurrent and repeated sleep and wake cycles [9]. As illustrated in Fig. 1, SDCP schedules the communication windows in a periodic pattern covering the entire alert period, during which the LLPMs are sensing and responding to intrinsic cardiac activity. While SDCP maintains AV-synchrony even for maximum beat-to-beat heart rate accelerations, it results in suboptimal energy-efficiency at typical heart rates and variability encountered during the majority of time. Specifically, the number of idle windows per cardiac cycle increases at lower heart rates, leading to a proportional increase in the idle current consumption for the ventricular receiver [9].

This work proposes an extension of SDCP based on a rate-responsive window scheduling algorithm (RR-SDCP), which dynamically adapts the frequency of communication windows to the momentary heart rate to minimize idle receiver current consumption. The power-saving potential of RR-SDCP and the resulting impact on AV-synchrony are analyzed as function of key control parameters to derive an optimal performance trade-off. The analysis is done based on simulations with human electrocardiogram (ECG) signals derived from the MIT-BIH Arrhythmia Database.

## Materials and methods

Section 2.1 describes the operation of SDCP, which is the basis of the proposed RR-SDCP presented in Sect. 2.2. The subsequent Sects. 2.3–2.5 describe the simulation setup, the input data preparation and the data analysis that was employed for the performance evaluation of RR-SDCP, respectively.

### Synchronous duty-cycling communication protocol: SDCP

Figure 1 illustrates the communication protocol implemented in this work, which is based on the concept of synchronous transceiver duty-cycling outlined in [9]. The main principle is to restrict communication to short periodic windows, allowing the LLPMs to save power by turning off their transceivers in the intervening sleep periods. The window interval during the alert period of the LLPMs is equal to the sensed AV-delay $t_{avd,s}$. More frequent communication is not required from a physiological point of view. During the refractory period following ventricular activation, the window interval is temporarily increased to an integer multiple of $t_{avd,s}$. Inter-device communication is organized based on a request-response protocol, with the ALLPM and the ventricular LLPM (VLLPM) as the primary and subordinate devices, respectively. A communication cycle is thus always initiated by a request of the ALLPM and is followed by the response of the VLLPM. In detail, the ALLPM sends a pacing trigger and the corresponding pacing delay $t_{pace}$ to the VLLPM in the next communication window after a sensed or paced P-wave. The VLLPM immediately responds in one of two ways, depending on whether it detected a late-cycle premature ventricular contraction (PVC) in the previous sleep period. In the more typical case without late-cycle PVC, the pacing command is simply acknowledged and executed. In the second case, the VLLPM responds by rejecting the pacing command to prevent pacing onto the T-wave. In addition, the VLLPM adds the elapsed time that has passed since the detection of the PVC to the response message. This enables the ALLPM to retrospectively reset its lower-rate counter such that the next pacing pulse would be delivered after a preset time delay relative to the PVC, which is the typical PVC response of conventional dual-chamber PMs [10]. In this work, a PVC is considered as late-cycle if the next sensed or paced P-wave occurs within a timeframe shorter than the post-ventricular atrial refractory period (PVARP), which is a fundamental setting of dual-chamber PMs [10].

### Rate-responsive window scheduling: RR-SDCP

**Fig. 2:**
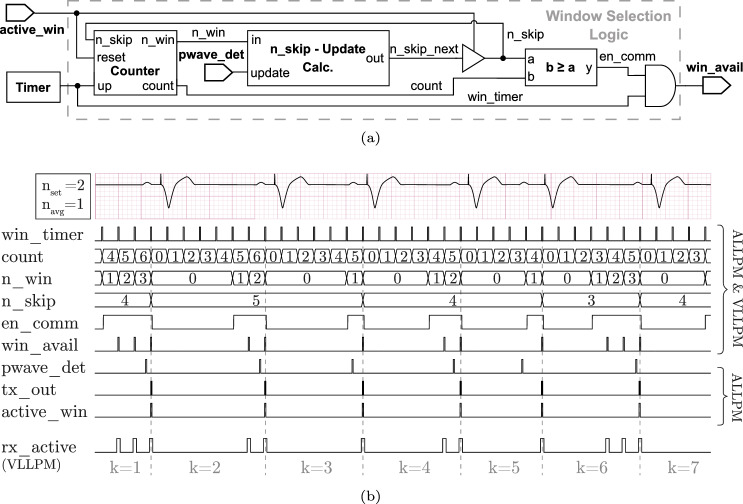
**a** Block diagram implementation and **b** timing diagram of rate-responsive window scheduling algorithm (RR-SDCP)

The goal of the RR-SDCP algorithm is to provide a fixed number of available communication windows $n_{set}$ per cardiac cycle. This requires that the sleep interval after an active window is dynamically adjusted based on the momentary heart rate.

Figure 2a shows the block diagram of the proposed window scheduling algorithm. A timer generates periodic pulses at the minimum required window interval given by $t_{avd,s}$, which are processed by the subsequent window selection logic (WSL). The WSL decides for each timer pulse whether the rest of the communication system is activated, i.e. if there is an available communication window.

As illustrated in Fig. 2b, the WSL follows a cyclic operation, where cycle boundaries are marked by active communication windows. In each cycle, the first $n_{skip}$ timer pulses do not result in available communication windows, referred to as skipped windows. After that, communication windows are made available at an interval of $t_{avd,s}$ until inter-device communication occurs and the cycle is complete. During each cycle, the state variable $n_{skip}$ is updated based on the difference $\Delta n_{win}[k]=n_{win}[k]-n_{set}$, where $n_{win}[k]$ represents the number of available windows in cycle *k* and $n_{set}$ denotes the target value. The update algorithm tries to regulate $\Delta n_{win}[k]$ to zero, by adjusting $n_{skip}$ in the next cycle accordingly, i.e. $n_{skip}[k+1]=n_{skip}[k]+\Delta n_{skip}[k]$. The update is thereby given by

1 $$\begin{aligned} \Delta n_{skip}[k] = {\left\{ \begin{array}{ll} -1 &{} \Delta n_{win}[k]<0, \\ 0 &{} \Delta n_{win}[k]=0, \\ s_{up}[k] &{} \Delta n_{win}[k]>0, \\ \end{array}\right. } \end{aligned}$$

where the positive increment $s_{up}[k]$ is calculated by averaging the last $n_{avg}$ cycles according to,

2 $$\begin{aligned} \begin{aligned} s_{up}[k]&=\left\lfloor \frac{1}{n_{avg}}\sum \limits _{i=0}^{n_{avg}-1} sign(\Delta n_{win}[k-i]) \right\rfloor \\&= {\left\{ \begin{array}{ll} +1 &{} \Delta n_{win}>0 \text { for all of the last } n_{avg} \text { cycles},\\ 0 &{} \text {otherwise} \end{array}\right. } \end{aligned} \end{aligned}$$

While negative deviations of $n_{win}$ from the setpoint immediately decrement $n_{skip}$ in the next cycle, positive deviations need to consistently persist over $n_{avg}$ cycles, before $n_{skip}$ is incremented. This asymmetry (assuming $n_{avg}>1$) reflects the heart rate profile during intense physical activity, where heart rate quickly increases at the onset of exercise but decelerates more slowly after activity ends [10].

To ensure overlapping communication windows, $n_{skip}[k]$ must be synchronized between the two LLPMs. The ALLPM calculates the update $\Delta n_{skip}$ immediately after detecting a P-Wave and appends it to each pacing request message. This approach only increases the message size by a maximum of two bits, while allowing the VLLPM to infer $n_{skip}[k]$ by accumulating updates.

### Simulation model

The performance of the RR-SDCP algorithm was analyzed by system-level simulations in Simulink (MathWorks, USA), based on functional models of the relevant LLPM components. The pacing units were modeled by implementing the dual-chamber timing cycles listed in Table 1 [10]. Each model featured a sense input for detecting the intrinsic electric activity of the respective cardiac chamber and a pacing output for stimulation. Inputs and outputs were were represented as 1-bit digital signals. The ALLPM model featured an additional mode_switch input. When set to logic high, the system effectively operates as a single-chamber ventricular pacemaker, i.e. the ALLPM will both, withhold pacing and not trigger ventricular pacing in response to a sensed atrial event.

The two LLPMs synchronize their activity by exchanging communication messages according to RR-SDCP outlined in Sects. 2.1 and 2.2. Each message consists of two header bits, specifying the type of the request or response message and a variable-sized payload. Table 2 lists the message types and their payload size $n_{b,pld}$. The last column depicts the total number of bits $n_{b,tot}$ required for actual physical data transmission. Thereby, $n_{b,tot}$ was calculated by adding $n_{b,pld}$ to an estimated number of eight overhead bits, including the message header and additional bits for packet synchronization, preamble and parity information.

For comparison to existing LLPMs, functional models of the AVEIR™ DR and the Micra™ AV were implemented based on their device manuals [7, 11]. The main difference of the AVEIR™ DR, which is also a dual-chamber system, is that inter-device communication is possible at any time. Consequently, a sensed P-Wave or PVC is instantaneously communicated by the ALLPM or VLLPM, respectively, to start the corresponding timing cycle in the other device. The Micra™ AV, a single-chamber ventricular LLPM, achieves AV-synchrony through remote mechanical sensing of the atrial contraction, which occurs with a delay compared to electrical sensing of the P-Wave in the atrium. [12, 13]. To account for this delay, the atrial sense input was delayed by 100 ms before being applied to the model [14].

The pacemaker settings for each model were set to the default values of the corresponding commercial device as listed in Table 1. The parameters for the RR-SDCP model were set to the same values as for the AVEIR™ DR.

**Table 1:** Implemented timing cycles and parameter values used in simulations

Parameter	Unit	Micra™	AVEIR™	SDC
Mode	–	VDD	DDD	DDD
Lower rate limit	bpm	50	50	50
Maximum tracking rate	bpm	115	130	130
Sensed AV-delay	ms	20	150	150
Paced AV-delay	ms	N/A	200	200
Safety pacing delay	ms	N/A	120	120
Post ventricular atrial				
refractory period (PVARP)	ms	550	275	275
Ventricular blanking period	ms	N/A	48	48
Crosstalk sensing window	ms	N/A	64	64
Ventricular refractory period	ms	240	250	250
Atrial refractory period	ms	N/A	250	250
Auto mode switch (AMS) mode	–	N/A	VDI	VDI
AMS base rate	bpm	N/A	80	80

**Table 2:** Message types exchanged between the two LLPMs and the number of bits $n_{b,pld}$ and $n_{b,tot}$ required to represent the payload and the complete message, respectively

Message type	SDCP	RR-SDCP	$\mathbf {n_{b,pld}}$	$\mathbf {n_{b,tot}}$
ALLPM $\xrightarrow {\phantom{a}}$ VLLPM (request)				
Pacing trigger	x		6	14
Mode switch	x	x	4	12
Pacing trigger w/o $n_{skip}$ update		x	7	15
Pacing trigger w/ $n_{skip}$ update		x	8	16
VLLPM $\xrightarrow {\phantom{a}}$ ALLPM (response)				
Acknowledge	x	x	0	8
PVC detection	x	x	7	15

### Data preparation

The sensing input for the simulation model, i.e. intrinsic electrical activity of the heart, was derived from human ECG recordings of the MIT-BIH arrhythmia database, freely available through PhysioNet [15, 16]. Each record in [15] provides QRS-complex annotations obtained from MLII and V1 (or V2/V5 for certain records) ECG leads. The selected subset included all 12 recordings with available P-wave annotations through [17]. Each beat annotation timeseries was transformed into a digital signal by generating a unit-duration pulse at the specified time for each annotated beat. To represent a typical pacemaker scenario, a complete AV-block was artificially induced, by removing all normal QRS-complexes from the ventricular channel. Beats labeled as PVCs were retained, as these may commonly occur in pacemaker patients. The mode_switch input of the ALLPM was derived from the rhythm annotations. Specifically, mode_switch was set to 1 during episodes of supraventricular tachycardias (SVTA) and to 0 during all other rhythms. Table 3 summarizes the characteristics of the input data.

**Table 3:** Signal statistics of ECG records from MIT-BIH arrhythmia database that were used as simulation inputs

Record	# P-Waves	# PVCs	SVTA [%]$^{\textrm{a}}$
100	2257	1	0.0
101	1865	0	0.0
103	2084	0	0.0
106	1507	520	0.0
117	1534	0	0.0
119	1620	444	0.0
122	2475	0	0.0
207	1415	105	2.9
214	2001	256	0.0
222	1257	0	29.6
223	2099	473	0.0
231	1994	2	0.0
Total	22108	1801	2.7

${\phantom{a}}^\textrm{a}$Relative time duration of supraventricular tachycardias

### Data analysis

Data analysis was performed in Matlab R2022b (MathWorks, USA) based on the recorded simulation outputs (i.e. not derived from implanted devices), which included the two pacing outputs and the communication signals (active window, message type and payload).

#### AV-synchrony and communication resources

AV-synchrony was evaluated for each ventricular beat in the VLLPM alert period. A beat was considered as AV-synchronous if preceded by a P-wave within a time interval of 80–200 ms. The lower limit was motivated as there needs to be a minimum delay between atrial and ventricular activation for the atrial kick to effectively contribute to the cardiac output [10]. The upper limit matches the paced AV-delay, which is the longest expected AV-interval for proper dual-chamber operation with the settings in Table 1. The AV-synchrony ratio was then calculated as

3 $$\begin{aligned} R_{avs}=\frac{n_{q,avs}}{n_{q,tot}}, \end{aligned}$$

where $n_{q,tot}$ and $n_{q,avs}$ represent the number of total and AV-synchronous QRS-complexes, respectively.

Communication resources were evaluated by recording the average frequency of communication windows $f_{win}$ and the average number of bits transmitted per second $r_{b}$, given by

4 $$\begin{aligned} r_{b}=\frac{1}{t_{sim}}\sum _{i=1}^{n_{msg}} n_{b,i}, \end{aligned}$$

where $n_{b,i}$ is the number of bits contained in message *i* (cf. Table 2), $n_{msg}$ the total number of transmitted messages and $t_{sim}$ the total duration of the simulation run.

#### Transceiver current estimation

The transceiver current is the sum of the average currents of the receiver and the transmitter. The receiver current was estimated by combining the simulation results of the communication resources with the electrical performance of the receiver prototype shown in Table 4. This receiver design was specially developed for SDCP-based LLPM synchronization and implemented as an application-specific-integrated-circuit in earlier work by the authors [9]. The average receiver current was calculated as

5 $$\begin{aligned} I_{rx}=I_{rx,on}d_{rx}=I_{rx,on}\frac{1}{t_{sim}}\sum _{i}t_{win,i}, \end{aligned}$$

where $I_{rx,on}$ represents the current consumption in active mode, $d_{rx}$ the average duty-cycle and $t_{win,i}$ the duration of the *i*-th window. The window duration $t_{win,i}$ depends on whether a message was received in the given window and is calculated as follows: each window has a minimum duration of $t_{win,id}$ to guarantee a sufficiently long listening time for message detection. This idle window duration is imposed by the hardware implementation (cf. [9] for details) and was set to $t_{win,id}=400$ $\mu$s and $t_{win,id}=270$ $\mu$s for the VLLPM and ALLPM, respectively. In case of an incoming message, the minimum window length is increased by the message duration $t_{msg,i}$, i.e. $t_{win,i}=t_{win,id}+t_{msg,i}$, with $t_{msg,i}=n_{b,i}/f_{data}$, where $n_{b,i}$ is the number of received bits and $f_{data}$ the communication data rate.

Transmitter consumption is mainly influenced by the current level sent through the communication channel. It must be high enough to ensure the receiver detects the input signal above its sensitivity threshold. Thus, the average transmitter output current was approximated by,

6 $$\begin{aligned} I_{tx,o}=\frac{V_{sens,rx}}{G_{ch}R_{el,tx}}\frac{r_b}{f_{data}}, \end{aligned}$$

where $V_{sens,rx}$ is the receiver sensitivity (cf. Table 4), $G_{ch}$ the channel attenuation and $R_{el,tx}$ the transmitter inter-electrode impedance. Parameter values of $G_{ch}=-66$ dB and $R_{el,tx}=400~\Omega$ representing typical intracardiac characteristics were used [6, 8, 18, 19].

**Table 4:** Measured electrical characteristics of the receiver in [9] that were used for transceiver current estimation

Parameter	Value
Active current $I_{rx,on}$	40 $\mu \hbox {A}$
Sensitivity $V_{sens,rx}$	82 $\mu \hbox {V}$
Data rate $f_{data}$	100 kb/s
Minimum window duration $t_{win,id}$	400 $\mu \hbox {s}$ (VLLPM)
Minimum window duration $t_{win,id}$	270 $\mu \hbox {s}$ (ALLPM)

## Results

**Fig. 3:**
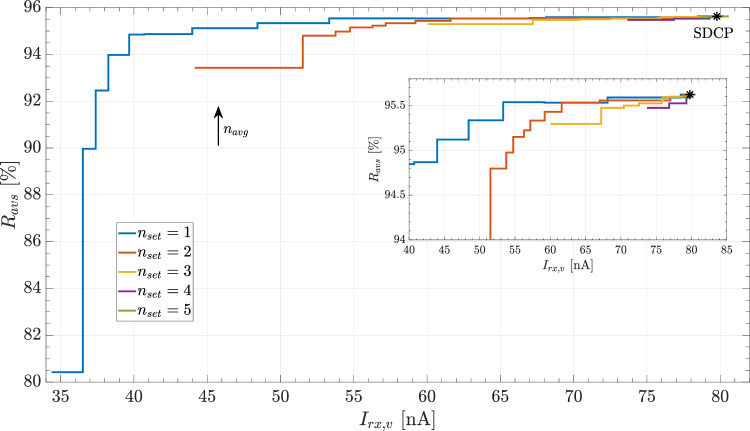
AV-synchrony and current consumption of ventricular receiver as function of the target number of windows per cardiac cycle $n_{set}$ and the averaging factor of the update algorithm $n_{avg}$

**Fig. 4:**
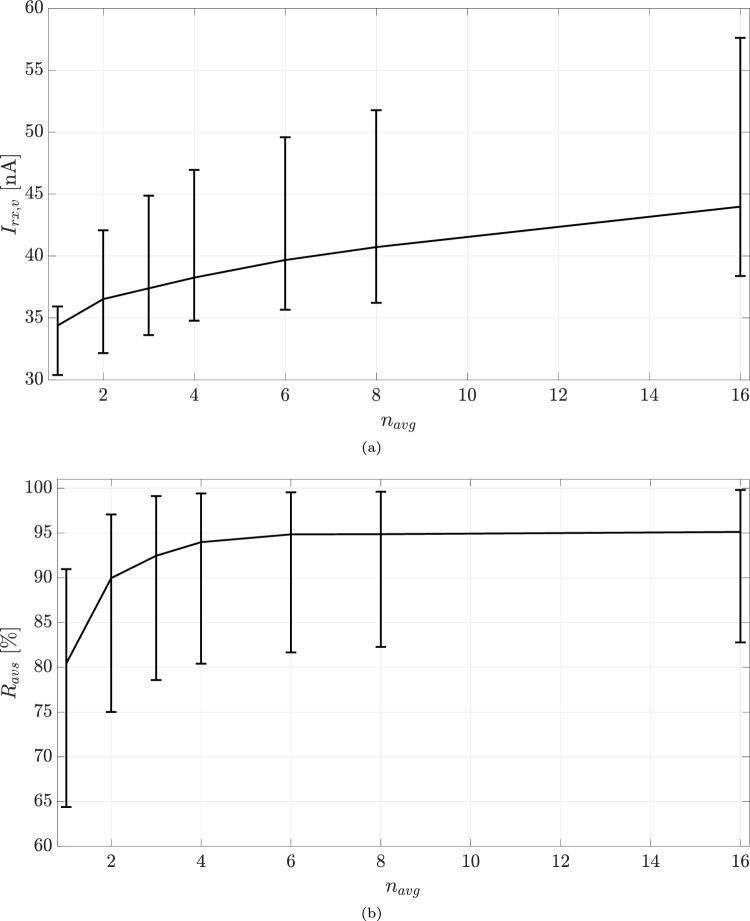
Median and IQR of **a** ventricual receiver current and **b** AV-synchrony as function of the averaging factor for $n_{set}=1$

Figure 3 shows the trade-off between the median values for AV-synchrony $R_{avs}$ and ventricular receiver current $I_{rx,v}$ as function of the target number of windows $n_{set}$ and the averaging factor $n_{avg}$. An increase in either $n_{set}$ or $n_{avg}$ individually increases both, $R_{avs}$ and $I_{rx,v}$. The pareto boundary between the points with minimum $I_{rx,v}$ and maximum $R_{avs}$ results for a fixed $n_{set}=1$ by varying $n_{avg}$.

Figure 4 plots the median and interquartile range (IQR) of $I_{rx,v}$ and $R_{avs}$ as function of $n_{avg}$ for $n_{set}=1$. The maximum value for the $R_{avs}/I_{rx,v}$-ratio is thereby achieved for $n_{avg}=3$, resulting in a median receiver current of $I_{rx,v}=37.4~$ nA (IQR $33.6-44.9$ nA) and a median AV-synchrony of $R_{avs}=92.5\%~(78.6-99.1\%)$. In contrast, base SDCP without rate-responsive window scheduling, achieves a median receiver current of $I_{rx,v}=79.8~$ nA ($77.2-81.3$ nA) and a median AV-synchrony of $R_{avs}=95.6\%~(83.3-100\%)$.

**Fig. 5:**
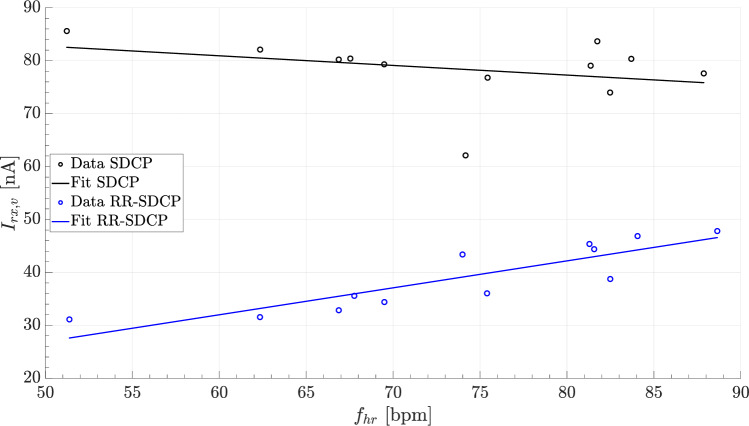
Ventricular receiver current versus average heart for each record

**Fig. 6:**
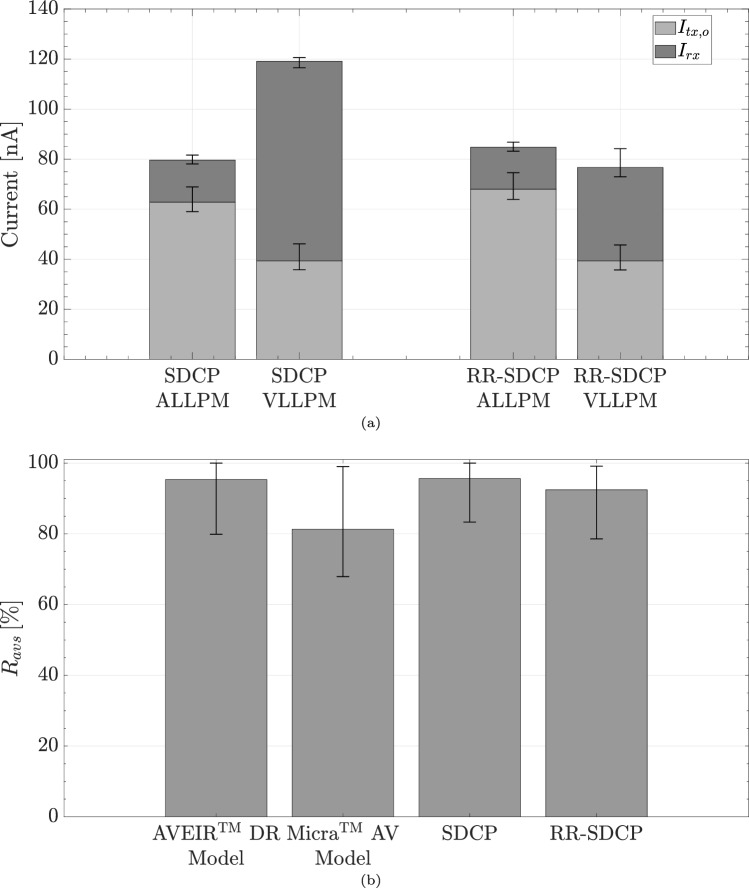
**a** Comparison of current consumption for complete dual-chamber LLPM system. **b** Comparison of AV-synchrony performance of SDCP and RR-SDCPP to simulation models of commercial LLPMs

Figure 5 shows a scatter plot of $I_{rx,v}$ against the average heart rate $f_{hr}$ for each individual record. As indicated by the fitted curves, RR-SDCP results in a positive linear correlation of $I_{rx,v}$ on $f_{hr}$, while an inverse and weaker correlation between $I_{rx,v}$ and $f_{hr}$ is observed for SDCP. Consequently, the relative reduction in $I_{rx,v}$ due to rate-responsive window scheduling increases with lower heart rates.

The total current consumption per device $I_{tot}$, given by the sum of the receiver current $I_{rx}$ and the transmitter output current $I_{tx,o}$, is shown in Fig. 6a. The overall system current, given by summing $I_{tot}$ of the ALLPM and the VLLPM, was estimated at a median value of 203 nA ($190-211$ nA) for SDCP and reduced to a median value of 166 nA ($152-183$ nA) for RR-SDCP with $n_{set}=1$ and $n_{avg}=3$.

Lastly, Fig. 6b compares AV-synchrony performance of SDCP and RR-SDCP to the simulation results for the AVEIR™ DR and Micra™ AV LLPM models, which achieved median values of $R_{avs}=95.4\%~(79.9-100\%)$ and $R_{avs}=81.3\%~(67.9-99\%)$, respectively.

## Discussion

This work analyzed the power-saving potential and the resulting impact on AV-synchrony for a novel rate-responsive dual-chamber LLPM communication protocol.

### Optimal RR-SDCP setting

The optimal trade-off between minimizing the ventricular receiver current and maximizing AV-synchrony is achieved with a target of one communication window per cardiac cycle, i.e. $n_{set}=1$ (cf. Fig. 3). On the one hand, larger values for $n_{set}$ result in a pareto-inefficient solution independent of the averaging factor $n_{avg}$. On the other hand, the resulting loss in $R_{avs}$ with $n_{set}=1$ can be kept within limits by choosing $n_{avg}$ sufficiently large. The most effective trade-off results from an $n_{avg}$ value between three and eight. Specifically, a moderate decrease in $R_{avs}$ of 1–3% enables a substantial reduction in $I_{rx,v}$ of 49-53% compared to the corresponding baseline values obtained with SDCP. Decreasing $n_{avg}$ below the indicated range to further reduce $I_{rx,v}$ becomes less favorable, as $R_{avs}$ starts to decrease at a similar rate (cf. Fig. 4).

The sub-100% AV-synchrony rates for non-rate-responsive SDCP and the AVEIR™ DR in Fig. 6b were caused by the presence of PVCs and SVTAs in the input data (cf. Table 3) rather than by any limitations in the LLPM simulation models.

Any degradation in AV-synchrony is expected to decrease cardiac output, since the atrial contribution to ventricular filling reduces [10]. Consistent AV-synchronous pacing enhances cardiac output on average by 15–25% compared to non-AV-synchronous ventricular pacing [10]. Thus, adopting RR-SDCP with $n_{set}=1$ and $n_{avg}\in [3,8]$, is expected to reduce cardiac output by $<1\%$ compared to SDCP or the communication protocol used by the AVEIR™ DR, since these parameter values resulted in a degradation of $R_{avs}$ of $<3\%$. This reduction in cardiac output is unlikely to notably affect the efficacy of pacemaker therapy or result in additional symptoms.

Nevertheless, the optimal trade-off between AV-synchrony and current consumption, and thus the optimal setting for $n_{avg}$, might vary across patients, depending on the native rhythm and therapy requirements. For example, patients with a high PVC burden, that also reduces AV-synchrony, might tolerate comparatively smaller additional losses in $R_{avs}$. Thus, if RR-SDCP is to be implemented in future LLPMs, $n_{avg}$ would ideally be realized as a programmable setting, which could be set patient- and therapy-specific by the physician.

### Implications for dual-chamber LLPM longevity

The ultimate goal of the proposed protocol is to increase the longevity of dual-chamber LLPMs by reducing the current consumption of inter-device communication. While this simulation study cannot precisely quantify the battery-life extension resulting from the protocol’s adoption, it allows for a reasonable estimation. Specifically, the major contributors to the transceiver current, namely the receiver front-end and the transmitter output current, were determined using realistic performance data from communication hardware specially developed for low-power LLPM synchronization (cf. Sect. 2.5.2). Multiplying the sum of the receiver and transmitter output current reported in Fig. 6a with an uncertainty factor of two, yields a conservative estimate for the total transceiver current of 140–160 nA per device. In comparison, the AVEIR™ dual-chamber LLPM is estimated to have a transceiver current consumption of 800 nA per implant under default settings [7]. Depending on the patient-specific pacing parameters, this synchronization current causes a reduction in the projected battery lifetime of 35–45% per device [7]. Thus, the above estimate indicates that the adoption of RR-SDCP may increase the longevity of future LLPMs by up to 25–35%, necessitating substantially fewer device replacements. The power-saving benefits of RR-SDCP may be particularly relevant in case of unfavorable pacing parameters that further reduce battery life in addition to implant communication, such as low pacing impedance, high pacing burden, and elevated pacing thresholds. The latter can occur, for example, in patients undergoing LLPM implantation after transvenous lead extraction due to PM-related infections [20]. Finally, reducing the number of replacement procedures would be highly beneficial from a clinical point of view, since device implantation represents one of the costliest and complication-prone steps in leadless pacemaker therapy [21].

### Limitations

The findings of this study have to be seen in light of some limitations. A complete analysis of the ramifications of the proposed protocol is only possible based on extensive in-vivo studies with dedicated hardware that implements the full dual-chamber LLPM system, which were beyond the scope of this study. However, the current work allows for a reasonable estimation of the relative changes in current consumption and AV-synchrony resulting from adopting a rate-responsive communication strategy. Specifically, heart rate variability, which is the most fundamental influencing factor, was realistically modeled based on a relatively diverse set of human ECG recordings. In addition, the transceiver current was estimated with electrical measurements of microelectronic hardware specially developed for the given application (cf. Sects. 2.5.2 and 4.2). Another limitation of this study is deriving the sensing inputs from surface ECG recordings, as LLPMs detect intracardiac electrograms (IEGMs) instead. Consequently, the absolute timing of the atrial or ventricular depolarizations detected by the LLPMs may slightly differ from the respective peaks in the surface ECG. However, RR-SDCP operates on PP-interval variations, which are independent of whether they are measured from ECG or IEGM signals. Thus, it is unlikely that the presented results would significantly change with IEGM-derived input signals.

## Conclusion

The results of this study suggest that adopting the proposed rate-responsive communication protocol may significantly reduce the current consumption of contemporary dual-chamber leadless pacemakers by 30 percent without causing a prohibitive loss in AV-synchrony. The implants’ lifespan could consequently be extended, potentially reducing the rate of expensive and complication-prone device replacements.

## Data Availability

This work contains information from the MIT-BIH Arrhythmia Database and the MIT-BIH Arrhythmia Database P-Wave Annotations which are made available under the https://opendatacommons.org/licenses/by/index.html ODC Attribution License. Data from both sources were used in compliance with the terms and conditions of the ODC Attribution License.
